# Exploring security threats and solutions Techniques for Internet of Things (IoT): from vulnerabilities to vigilance

**DOI:** 10.3389/frai.2024.1397480

**Published:** 2024-05-15

**Authors:** Swastik Kumar Sahu, Kaushik Mazumdar

**Affiliations:** Department of Electronics Engineering, IIT (ISM), Dhanbad, India

**Keywords:** threats and security in IoT, blockchain for IOT security, edge computing, FOG computing, machine learning, Twofish technology, Diffie-Hellman encryption technique

## Abstract

The rapid proliferation of Internet of Things (IoT) devices across various industries has revolutionized the way we interact with technology. However, this widespread adoption has also brought about significant security challenges that must be addressed to ensure the integrity and confidentiality of data transmitted and processed by IoT systems. This survey paper delves into the diverse array of security threats faced by IoT devices and networks, ranging from data breaches and unauthorized access to physical tampering and denial-of-service attacks. By examining the vulnerabilities inherent in IoT ecosystems, we highlight the importance of implementing robust security measures to safeguard sensitive information and ensure the reliable operation of connected devices. Furthermore, we explore cutting-edge technologies such as blockchain, edge computing, and machine learning as potential solutions to enhance the security posture of IoT deployments. Through a comprehensive analysis of existing security frameworks and best practices, this paper aims to provide valuable insights for researchers, practitioners, and policymakers seeking to fortify the resilience of IoT systems in an increasingly interconnected world.

## 1 Introduction

The Internet of Things (IoT) has emerged as a transformative technology paradigm, connecting a multitude of physical objects embedded with sensors and actuators to enable seamless communication and data exchange over the Internet. This interconnected network of “things” holds immense promise for revolutionizing industries such as healthcare, agriculture, transportation, and smart cities, offering unprecedented levels of efficiency and convenience (Al-Turjman and Lemayian, 2020). However, the widespread adoption of IoT devices has also exposed critical security vulnerabilities that pose significant risks to data privacy, system integrity, and overall network resilience. Highlighting the potential of IoT to drive innovation and enhance societal services, this paper delves into the multifaceted landscape of IoT security threats and challenges. From malicious cyber attacks targeting IoT devices to the exploitation of vulnerabilities in network protocols and data transmission mechanisms, the security risks facing IoT ecosystems are diverse and complex (Saračević et al., 2022). The escalating sophistication of cyber threats coupled with the proliferation of interconnected devices underscores the urgent need for robust security measures to safeguard sensitive information and mitigate potential risks. By elucidating the key problem statements surrounding IoT security, this paper aims to shed light on the critical importance of addressing these challenges to ensure the safe and reliable operation of IoT systems (Saračević et al., 2020). Through a comprehensive examination of existing security frameworks, emerging technologies, and best practices, we seek to provide valuable insights and practical solutions for enhancing the security posture of IoT deployments. By fostering a deeper understanding of the security implications inherent in IoT environments, we strive to empower stakeholders across academia, industry, and policymaking to proactively mitigate risks and fortify the resilience of IoT ecosystems in an increasingly interconnected world (Puthal et al., 2022).

## 2 Architecture of IoT

**Figure 1:**
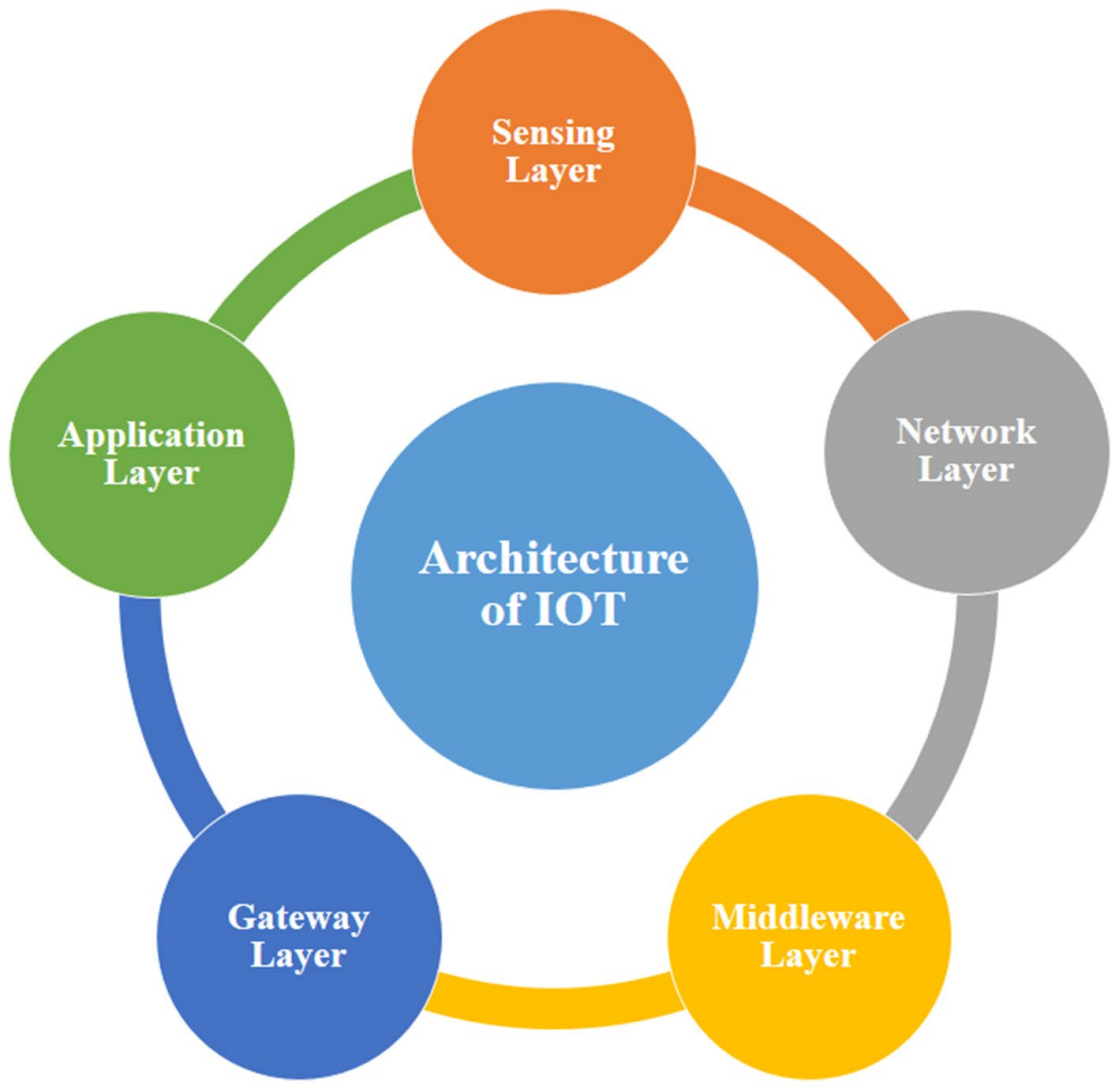
The architecture of the IoT framework.

Within the Internet of Things (IoT) framework, each layer is characterized by its functions and the devices employed within that layer. While there are varying perspectives on the number of layers in IoT, many researchers (Hassija et al., 2019) generally agree on a five-layer model. Those five layers are the Sensing Layer, Network Layer, Middleware Layer, Gateway Layer, and Application Layer which are represented in Figure 1. In the implementation of IoT, each of these layers leverages diverse technologies, giving rise to various challenges and security threats. It is essential to recognize that the interaction and integration of technologies across these layers contribute to the overall functionality and effectiveness of an IoT system.

## 3 Issues of IoT security

### 3.1 Sensing layer security issues

The Sensing Layer in IoT is intricately linked with physical sensors and actuators, where sensors detect the physical phenomena in their surroundings, and actuators execute tasks based on the information gathered by these sensors (Jing et al., 2014). A variety of sensors, such as ultrasonic sensors, camera sensors, smoke detection sensors, temperature and humidity sensors, etc., are employed to collect different types of information. These sensors can find applications in various IoT scenarios like GPS, RFID, RSNs, WSNs, etc. However, the Sensing Layer is vulnerable to several security threats:

- *Sensors tampering:* Adversaries may target sensors and actuators in IoT applications, gaining control over them. This unauthorized interference can lead to a complete failure of the IoT application (Pathak et al., 2021).
- *Sending false code:* Adversaries may inject false information into the memory of sensors. As firmware or software updates for IoT nodes often occur wirelessly, this creates an opportunity for adversaries to send malicious code. This false code can coerce sensors into performing unintended actions or compromise the entire IoT system, potentially causing a Distributed Denial of Service (DDoS) attack (Jazzar and Hamad, 2022).
- *Side-channel attacks (SCA):* SCA, relying on electromagnetic attacks, power consumption analysis, laser-based attacks, and timing attacks, can leak critical information. Implementation of cryptographic modules can help prevent such attacks (Zankl et al., 2021).
- *Eavesdropping and interference*: Sensors, often deployed in open environments, are susceptible to tampering and information capture during data transmission and authentication processes by adversaries (Anajemba et al., 2022).
- *Increasing power consumption:* Attackers might manipulate IoT edge devices by introducing false code or running infinite loops, causing a surge in power consumption. This can lead to the rapid depletion of batteries, resulting in a service denial response because of dead batteries (Goudarzi et al., 2021).

### 3.2 Network layer security issues

The Network Layer plays a crucial role in transmitting sensor data from the Sensing Layer to the server for processing in an IoT environment (Sharma et al., 2017). However, this layer is susceptible to various security issues:

- *Phishing site attack:* Adversaries may execute phishing attacks by sending deceptive websites to users to extract their account credentials. Once malicious actors obtain this valuable information, they can assert control over the entire IoT application (Alkhalil et al., 2021).
- *DDoS/DoS attack:* Attackers disrupt services for legitimate users by overwhelming target servers with an extensive volume of requests. The Mirai botnet, for example, exploited this vulnerability by constantly bombarding weakly configured IoT devices, leading to the blockage of various servers (Bårli et al., 2021).
- *Routing attacks:* In an IoT setup, invaders may attempt routing attacks during information transportation. Sinkhole attacks involve diverting sensing requests from a falsely beneficial routing path, attracting numerous nodes to direct traffic through it. While this attack may not directly disrupt network function, when combined with additional attacks, it can develop a potent application. A wormhole attack, which is another manifestation of a routing attack, presents a substantial security threat. Wormhole attacks entail establishing a tunnel between a compromised node and an internet-connected device, aiming to circumvent fundamental security protocols in an IoT application. The challenge in detecting this attack lies in its capacity to observe network actions without causing alterations (Agiollo et al., 2021).

### 3.3 Middleware layer security issues

The Middleware Layer functions as a vital link between the Network and Application Layers in IoT, delivering computing and storage capabilities while furnishing APIs to fulfill the requirements of the Application Layer (Zhang et al., 2021). Comprising components plays a pivotal role. Nonetheless, it is not impervious to attacks, and various techniques can jeopardize the entire IoT application. Key security challenges encompass issues related to database security and the security of cloud servers. The list of middleware attacks includes:

- *Man-in-the-middle attack:* If adversaries gain unauthorized access to the broker and assume a man-in-the-middle position, there exists a potential risk of them taking control of the entire IoT application.
- *SQL sending attack*: The Middleware Layer is susceptible to SQL Injection (SQLi) attacks, where adversaries send false SQL statements to a program. This can result in the retrieval of secret information from the client and potential alterations to data in the cloud.
- *Signature wrapping attack:* Attackers may use XML signatures to execute signature wrapping attacks. In this method, adversaries manipulate the signature algorithm and execute false data by sending SOAP (Simple Object Access Protocol).
- *Sending cloud malware*: Adversaries may endeavor to gain control by injecting counterfeit code or virtual machine instructions into the cloud. By masquerading as a legitimate service, they could create a virtual machine instance or a deceptive service module, thereby potentially capturing sensitive information.
- *Flooding attack in cloud:* Similar to a Denial of Service attack, a flooding attack in the cloud affects the Quality of Service (QoS) by continuously sending multiple requests to a service. The objective of this attack is to exhaust cloud resources, deliberately escalating the load on the cloud servers.

### 3.4 Security issues at the gateway

The Gateway Layer plays a crucial role in connecting users and cloud services in the IoT architecture. It provides both hardware as well as software solutions for IoT devices, handling the encryption and decryption of information and managing protocols across different layers. However, this layer is not immune to security threats, and several gateway attacks are possible:

- *Secure on-boarding:* The Gateway Layer, which acts as an intermediate between users and managing services, is critical in ensuring safe data transmission. Nonetheless, it is vulnerable to man-in-the-middle attacks and key tampering, particularly during the onboarding process.
- *End-to-end encryption:* Ensuring end-to-end encryption is crucial for security in the Application Layer. The implementation should be designed to prevent unauthorized decryption by third parties, maintaining the confidentiality and integrity of the transmitted data.
- *Firmware updates:* Gateways play a critical role in downloading firmware updates, and it is imperative to establish a secure process for this task. Maintenance of records for new firmware versions and validation of signatures during the download of firmware updates are essential security measures. This helps prevent the installation of malicious or unauthorized firmware, ensuring the security and integrity of the IoT devices connected through the gateway.

### 3.5 Security issues at the application layer

**Figure 2:**
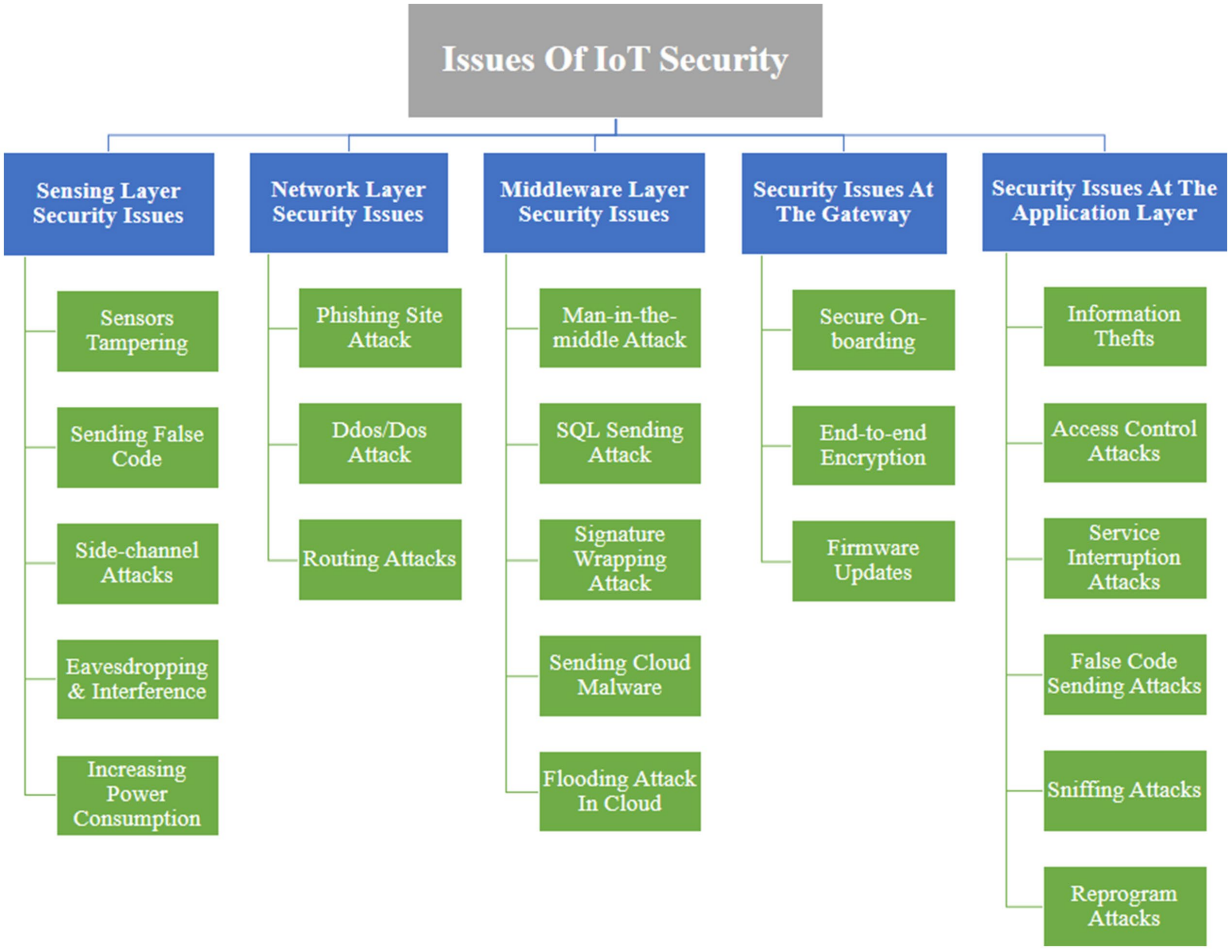
Issues of IoT securities.

The Application Layer, as the end-users layer, is in charge of offering services to users across a variety of domains such as smart homes, smart meters, smart cities, smart grids, and so on. However, this layer is susceptible to several attacks as mentioned in Figure 2.

- *Information thefts*: Users often store private information in IoT applications, making them vulnerable to information threats. To mitigate information theft, various methods and protocols like encryption, information isolation, client and network authentication, and privacy management can be employed.
- *Access control attacks:* Access control is a critical authentication method for users to access account information. If access control is compromised, attackers can gain control over the entire IoT application, posing a significant threat to security.
- *Service interruption attacks:* In service interruption attacks, users receive a busy response while attempting to access IoT applications, denying authentic users proper services.
- *False code sending attacks:* Adversaries may use Cross-Site Scripting (XSS) to send false data to a trusted website, potentially compromising the IoT account and tampering with the IoT system.
- *Sniffing attacks:* Attackers may utilize sniffer applications to track network traffic in IoT applications. Without proper security protocols, adversaries can obtain client secret information from the application.
- *Reprogram attacks:* If the programming procedure is not effectively secured adversaries may attempt to rewrite the secret code. This can cause the entire IoT system to malfunction. To prevent such attacks, it is critical to implement strong security measures during the programming process.

## 4 IoT security solutions

**Figure 3:**
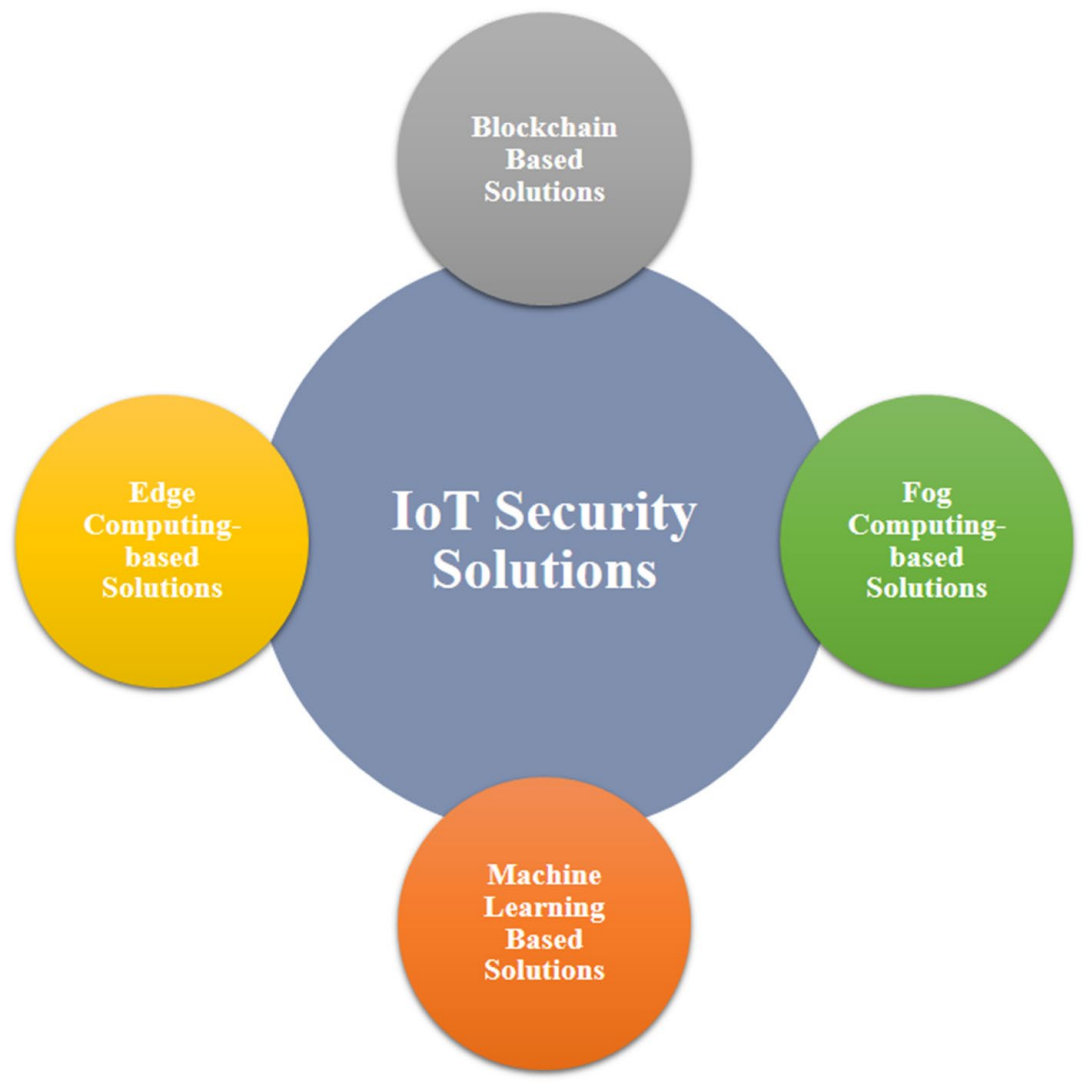
IoT security solutions.

To secure IoT environments and applications there are various methods *viz.*: blockchain-based solutions, fog computing-based solutions, machine learning-based solutions and edge computing-based solutions (Hassija et al., 2019). These methods have been illustrated in Figure 3.

### 4.1 Blockchain for IoT security

Blockchain plays a crucial role in bolstering security within the realm of IoT. This technology significantly enhances overall transparency, visibility, and levels of ease and trust for users. Blockchain, which uses a distributed, decentralized, and shared ledger, is an important component in assuring information security. It operates as a distributed ledger, with data entries organized chronologically and time-stamped. Every data point in the ledger is securely linked to its predecessor through cryptographic hash keys. Furthermore, the utilization of a Merkle tree allows for the storage of private transactions.

#### 4.1.1 Permissioned and permission-less blockchain

The architecture of blockchains can be categorized into two types based on information characteristics and implementation methods: permissioned and permission-less blockchains. The distinctive feature of permission-less blockchain lies in the fact that joining this network does not require any special permission. Bitcoin exemplifies a permission-less blockchain where participants can freely join or leave the network. While this type of blockchain can upkeep a maximum number of nodes, its fan-out is relatively lower. In contrast, permissioned blockchains operate under a defined set of rules that participants must adhere to join the network. Examples of permissioned blockchains include Ripple and Hyperledger. Permissioned blockchains generally have a higher fan-out compared to permission-less ones.

#### 4.1.2 Blockchain benefits

The blockchain has various advantages in IoT.

- *Storing IoT device information in blockchain:* IoT applications encompass a diverse array of interconnected devices, mutually measured and linked. This network extends into the fog, enabling versatile use of IoT applications. Given the expansive space for information transfer, blockchain emerges as an adept solution for safely storing and transmitting information, safeguarding it from unauthorized alterations.
- *Secure information storage through blockchain:* The decentralized nature of blockchain architecture mitigates the risk associated with single points of failure, a vulnerability often found in numerous fog-based IoT applications. Regardless of the geographical distance between devices, blockchain offers a secure means of storing information (Mahmoud et al., 2015).
- *Information encryption using hash keys*: Within the realm of blockchain, only the 256-bit hash key of the information is preserved before storing the original data. The true information can then be saved in the cloud, accompanied by the documented hash key. Altering the information involves changing the hash, ensuring security and isolation. The blockchain’s length remains unaffected by information size, as only hash values populate the chain. Honest clients can access cloud-stored information using the hash, with each data set authenticated by another client in the network, reducing the likelihood of unethical information storage.
- *Prevention of information loss and spoofing attacks:* Blockchain serves as a deterrent against spoofing attacks in IoT applications, where adversary nodes attempt to infiltrate and replicate within the network. The registration of authentic clients or devices on the blockchain facilitates easy identification and authentication without relying on certification authorities. Due to the low-power nature of IoT devices, blockchain prevents information loss by making additions to the chain irreversible.
- *Prevention of unauthorized access through blockchain:* Many IoT applications necessitate daily communication with clients, and blockchain establishes communication channels using private and public keys. Only the intended recipient can access the encoded information, enhancing security and addressing safety concerns prevalent in IoT applications.
- *Proxy-based architecture of blockchain:* Despite blockchain’s inherent security features for distributed environments, IoT faces resource constraints. Proxy-based architecture emerges as a promising solution, allowing IoT devices to leverage blockchain without the burden of storing large ledgers. Proxy servers, which are placed around the network, hold encrypted content that clients can download.
- *Elimination of centralized cloud servers:* Blockchain contributes to enhanced IoT system security by eliminating centralized cloud servers, and transitioning the network to a peer-to-peer model. This decentralization and encryption using cryptographic hash functions reduce the vulnerability of centralized cloud servers, often targeted by information thieves. Information is distributed across all network nodes, further fortifying security.

#### 4.1.3 The Merkle tree

The Merkle tree serves as an augmentation to the blockchain information structure, providing a heightened level of security for IoT devices. Moreover, it aids in decreasing the overall quantity of blocks appended to the chain. This approach effectively reduces the number of blocks in the blockchain. The use of multiple levels of hashing within the Merkle tree enhances the security of information at every level, further fortifying the integrity of the data. Given the frequent small-scale communications among IoT devices, incorporating the Merkle tree alongside blockchain emerges as a promising solution. This integration not only enhances security but also streamlines the structure of the blockchain, making it more efficient for the specific communication patterns characteristic of IoT devices.

#### 4.1.4 IOTA

IOTA stands out as a promising and innovative solution, serving as a highly auspicious key for securing IoT. Operating as a Distributed Ledger Technology (DLT) comparable to blockchain, IOTA distinguishes itself by specifically addressing the challenges posed by resource-constrained IoT applications. Every incoming request within the system is mandated to authenticate the preceding two requirements. A noteworthy aspect of IOTA’s demand authentication strategy is the incorporation of a tip selection algorithm in its implementation. This algorithm assigns increasing weights to all needs, with a higher weight indicating added security for the corresponding nodes in the system. This strategy not only improves the security posture of each of the nodes but also improves the overall robustness of the IoT ecosystem. IOTA diverges from traditional blockchain structures by adopting a tangled information structure, in contrast to the restrictive information structure found in conventional blockchains. This distinction reflects IOTA’s innovative approach to handling and verifying transactions, making it particularly well-suited for the unique requirements and limitations of resource-constrained IoT applications.

### 4.2 IoT security by fog computing

#### 4.2.1 Cloud to fog evolution

IoT (Internet of Things) and cloud computing are distinct technologies, each offering a myriad of applications. IoT has significantly expanded the realm of smart devices and applications, enriching user experiences. Meanwhile, cloud computing provides an efficient solution for storing and managing information, ensuring accessibility from any location, and is widely adopted by numerous organizations. The proliferation of IoT has led to an unprecedented surge in data generation, imposing a considerable burden on Internet infrastructure. To address the challenges and seize new opportunities in processing, storing, managing, and securing information, the integration of cloud and IoT has become pivotal. This integration introduces a novel era with both prospects and challenges, prompting industry and research groups to devise solutions for the issues faced by IoT within the cloud environment. However, the benefits derived from the cloud and IoT integration alone prove insufficient to tackle all challenges. Recognizing this, Cisco introduced the concept of fog computing in 2012. Unlike replacing cloud computing, fog computing serves as a complementary approach. It aims to address specific challenges faced by IoT, offering a distributed and decentralized computing model that brings computational resources closer to the edge of the network. This proximity enables faster data processing, reduced latency, and enhanced efficiency, thereby complementing the capabilities of traditional cloud computing in the IoT landscape.

#### 4.2.2 The architecture of fog computing

The major function of fog computing is to manage the data generated by adjacent IoT devices for effective monitoring, necessitating a multi-layered architecture. There are two frameworks in fog computing: the Fog-Device framework and the Fog-Cloud-Device framework (Zhang et al., 2014). The first type is made up of device and fog layers, whereas the latter one is made up of device, fog, and cloud layers. These layers are organized based on their storage and computational capabilities. Communication between layers is accomplished via either wired methods (such as optical fiber or Ethernet) or wireless ways (such as Wi-Fi, Bluetooth, and so on). Fog nodes in the Fog-Device framework provide numerous services to clients without the involvement of cloud servers (Balevi and Gitlin, 2018). Basic decisions, on the other hand, are retained at the fog layer in the Fog-Cloud-Device framework, while complex decisions are deferred to the cloud.

#### 4.2.3 Advantages of fog computing

IoT devices generate substantial volumes of data with each operation, making real-time transmission to the cloud for analysis impractical. To address this challenge, the concept of fog computing has emerged, aiming to extend the capabilities of cloud computing to the network’s edge. Fog computing, characterized by a distributed architecture for data analysis and computation, efficiently handles time-sensitive information, enhancing security, preventing data leakage, and minimizing reliance on cloud storage to boost overall IoT application efficiency (Ni et al., 2017). The reduced latency in fog computation, compared to cloud computation, results from the proximity of the fog layer to devices. Only crucial and selected data is forwarded to the cloud for long-term storage. Fog computing finds applications in various domains, including smart vehicles, homes, agriculture, healthcare, traffic management, retail, and software-defined networks (Hu et al., 2017). Transmitting vast amounts of IoT-generated data to the cloud for processing is both costly and time-consuming. Fog nodes, which can be devices like routers, switches, or video surveillance cameras with computing, storage, and network connectivity, can be strategically placed, such as on a factory floor or within a vehicle, as long as there is a network connection. Furthermore, fog nodes enhance the security of communication in IoT applications by using cryptographic computations, a feature often lacking in basic sensors and IoT devices (Mukherjee et al., 2018).

#### 4.2.4 IoT security threats overcome by fog computing

The answer that fog computing gives or may offer for resolving those security issues is explained more below.

i. *Man-in-the-Middle Attack:*

Fog functions as a security layer positioned between the end client and the cloud or IoT system. Any attacks targeting IoT systems must traverse the fog layer, enabling the identification and mitigation of abnormal activities before reaching the system (Salem et al., 2021).

ii. *Information Transit Attacks:*

Optimal information storage and management occur when conducted on secure fog nodes, in contrast to IoT devices. Storing information on fog nodes enhances protection, ensuring that client information remains more secure and readily accessible (Liang and Kim, 2021).

iii. *Eavesdropping:*

By facilitating communication exclusively between the end client and the fog node, fog nodes minimize the need to route information through the whole network. This significantly reduces the likelihood of eavesdropping attempts by adversaries, given the decreased traffic on the network (Anajemba et al., 2022).

iv. *Resource-Constraint Issues:*

Numerous IoT devices grapple with limitations in resources, rendering them vulnerable to potential exploitation by adversaries. In response to this challenge, fog nodes play a crucial role in offering support to edge devices, shielding them from potential attacks. Moreover, the proximity of these fog nodes allows them to carry out more advanced security functions, thereby bolstering the overall system’s resilience against potential threats (Imteaj et al., 2021).

v. *Incident Response Services:*

Fog nodes possess the capability to be programmed to provide real-time incident response services. In instances where they come across suspicious information or requests, these fog nodes promptly generate alerts to the IoT system or end-users. The inherent feature of fog computing enables the detection of malware and the resolution of issues during data transit. Fog nodes play a pivotal role in facilitating resolutions while ensuring the continuous operation of the system (Chemodanov et al., 2020).

#### 4.2.5 Security challenges and solution in fog layer

While the fog layer adds several features and improves security for IoT applications, moving information and processing to this layer exposes new risks (Mukherjee et al., 2020). Before implementing fog-assisted IoT applications, a thorough assessment of the security and privacy objectives of fog computing is required. This section digs into the fog layer’s various aspects, investigates the privacy and security difficulties encountered, and suggests methods to properly solve these challenges.

##### 4.2.5.1 Real-time services

Fog computing aims to deliver nearly real-time services within IoT systems by executing computations in close proximity to the points where information is generated.

i. *Intrusion detection:*

Without a proper intrusion detection mechanism, policy violations, and false activities on fog nodes and IoT devices may go unnoticed (Verma and Ranga, 2020). Adversaries can potentially manipulate local services. Fog nodes can collaborate with neighboring nodes to detect attacks targeting local services. Monitoring the behavior of programs and host file systems allows for the detection of cloud attacks.

ii. *Identity authentication:*

Various organizations, such as fog nodes, service providers, and users, are involved in the process of providing and accessing real-time services. Establishing confidence in all parties involved is a huge problem, raising security risks for IoT services and customer data (Shukla et al., 2021). To prevent adversaries from compromising servers and exploiting services and client privacy, access to services should only be provided to real and reputable users. As a result, effective identity authentication procedures are required to ensure secure services. Several recommendations for strong identity authentication methods have been made in earlier times.

##### 4.2.5.2 Transient storage

Users can temporarily store and manage their information on fog nodes through transient storage. While it facilitates easy information management on local storage, it introduces new challenges and security issues, particularly concerning information privacy.

i. *Identifying and protecting sensitive information:*

The data saved in IoT devices includes a variety of information, such as social gatherings, conditions in traffic, private activities, and temperature. A few of this information may be private or highly sensitive, while others may be open to the public. Furthermore, the same material may have various security settings for different users. As a result, it is critical to detect and protect important information among the massive amounts of data.

ii. *Sharing information securely:*

Security measures entail encrypting information uploaded on fog nodes, making it readable only by its owner. However, this encryption poses challenges for information sharing. These methods aim to enable secure and controlled sharing of encrypted information.

##### 4.2.5.3 Information dissemination

The transfer of information to the fog node necessitates encryption because of security concerns. However, such encryption introduces challenges and compromises some desirable features like sharing, searching, and aggregation.

i. *Searching information securely:*

In keeping with the notion of transitory storage, information is encrypted before being uploaded. Searching or recovering information from encrypted ciphertext gets difficult for both owners as well as other entities. To address this issue, searchable encryption methods and their associated privacy levels are defined, providing a framework for securely retrieving information from encrypted text.

ii. *Information aggregation:*

To minimize information loss while decreasing communication overhead, fog nodes may need to aggregate information in some instances (Shen et al., 2020). To combat information theft, secure aggregation techniques must be developed. To achieve secure information aggregation, several homomorphic encryption techniques have been suggested (Doan et al., 2023). These schemes enable fog nodes to aggregate information while preserving the confidentiality of individual data points.

##### 4.2.5.4 Decentralized computation

Decentralized computations pose several risks. Adversaries have the potential to manipulate the analyzed results and expose processed information.

i. *Server-aided computation:*

Tasks beyond the capability of IoT devices can be performed with the assistance of fog nodes. However, this introduces a risk of information exposure to adversaries, especially If the fog nodes that acquired information from IoT devices get compromised. Server-aided computing is a way to ensure secured computing, aiming to mitigate the risks associated with processing sensitive information on fog nodes.

ii. *Verifiable computation:*

Clients rely on fog nodes for calculating their information, highlighting the necessity for a secure means to validate the fog node’s calculation results. Verifiable computation methods are essential to instill confidence in users that the computations performed by fog nodes are accurate and untampered. This ensures the integrity and reliability of the computation results.

#### 4.2.6 Integration of industry 4.0 and IoT

The Fourth Industrial Revolution, often referred to as Industry 4.0, is characterized by the integration of digital technologies into industrial processes, leading to the emergence of smart factories and intelligent manufacturing systems. At the core of Industry 4.0 lies the concept of interconnectedness, where machines, devices, and systems communicate and cooperate autonomously. In this paradigm, the Internet of Things (IoT) plays a pivotal role by providing the infrastructure for seamless connectivity and data exchange. IoT encompasses a network of interconnected devices equipped with sensors, actuators, and communication technologies, enabling them to collect, analyze, and exchange data. On the other hand, Industry 4.0 represents a holistic approach to industrial transformation, leveraging technologies such as artificial intelligence, big data analytics, and cyber-physical systems to create intelligent and adaptive manufacturing environments. The integration of IoT in Industry 4.0 involves leveraging IoT technologies to enhance various aspects of industrial processes, including monitoring, control, optimization, and decision-making. This integration aims to create smart, connected, and autonomous manufacturing systems capable of adapting to dynamic environments and fulfilling the requirements of Industry 4.0 (Mutluturk et al., 2021).

Below are the key components and aspects of IoT integration in Industry 4.0:

i. *Real-time monitoring and sensing:* IoT-enabled sensors and devices are deployed throughout the manufacturing environment to monitor various parameters such as temperature, pressure, humidity, vibration, and energy consumption in real time. These sensors collect vast amounts of data from equipment, machinery, and production lines, providing valuable insights into the performance and health of assets. For example, sensors embedded in machines can detect anomalies or deviations from normal operating conditions, enabling predictive maintenance to prevent costly breakdowns and downtime (Singh and Singh, 2023).
ii. *Process optimization and automation:* IoT integration enables dynamic process optimization and automation by leveraging real-time data insights to adjust production parameters, allocate resources, and optimize workflows. For instance, predictive maintenance alerts can trigger automated workflows to schedule maintenance tasks, order replacement parts, and reconfigure production schedules in response to equipment failures or maintenance requirements. Furthermore, IoT-enabled actuators and controllers can autonomously adjust process parameters based on predefined rules or optimization algorithms, maximizing efficiency and resource utilization (Mutluturk et al., 2021).
iii. *Supply chain visibility and traceability:* IoT enhances supply chain visibility and traceability by enabling the tracking and monitoring of goods, materials, and components throughout the production and distribution process. RFID tags, barcodes, and sensors attached to products enable real-time tracking of their location, condition, and status as they move through the supply chain. This visibility enables organizations to optimize inventory management, mitigate supply chain disruptions, and ensure compliance with regulatory requirements and quality standards (Fatorachian and Kazemi, 2021).
iv. *Quality control and assurance:* IoT integration facilitates real-time quality control and assurance by monitoring and analyzing production processes and product attributes. Sensors embedded in production equipment can detect defects, deviations, or anomalies in product specifications, triggering alerts or automated corrective actions to maintain product quality standards. Additionally, IoT-enabled inspection systems can capture and analyze images, videos, or sensor data to identify defects or anomalies during production, enabling timely intervention and quality assurance (Ammar et al., 2022).

Overall, the integration of IoT in Industry 4.0 enables organizations to create intelligent, connected, and adaptive manufacturing systems capable of optimizing performance, enhancing efficiency, and driving innovation in the digital era. By harnessing the power of IoT technologies, industries can unlock new opportunities for growth, competitiveness, and sustainability in the evolving landscape of Industry 4.0.

### 4.3 IoT security using Diffie-Hellman encryption technique

Using the Diffie-Hellman encryption technique for IoT (Internet of Things) security involves employing this method for secure key exchange between devices to establish a shared secret key. The Diffie-Hellman key exchange algorithm allows two parties to agree on a shared secret key over an insecure communication channel, without directly transmitting the key itself. This shared secret key can then be used for subsequent symmetric encryption of the communication between the devices (Quist-Aphetsi and Xenya, 2019).

Here’s a simplified overview of how Diffie-Hellman works:

- Key Exchange Initialization:
  - Two IoT devices, let us call them Device A and Device B, decide to establish a secure communication channel.
  - They agree on a public modulus (p) and a base (g), which are known to both parties.
- Private Key Generation:
  - Each device generates its private key.
  - Device A generates private key a and Device B generates private key b.
- Public Key Calculation:
  - Using the public modulus, base, and their respective private keys, both devices calculate their public keys.
  - Device A calculates A= $g^{a}\;\mathrm{mod}\;p$
  - Device B calculates B= $g^{b}\;\mathrm{mod}\;p$
- Key Exchange:
  - Devices exchange their public keys over the insecure channel.
  - Device A receives B from Device B, and Device B receives A from Device A.
- Shared Secret Key Calculation:
  - Each device calculates the shared secret key using the received public key and its private key.
  - Device A computes s=$B^{a}\;\mathrm{mod}\;p$
  - Device B computes s=$A^{b}\;\mathrm{mod}\;p$

**Figure 4:**
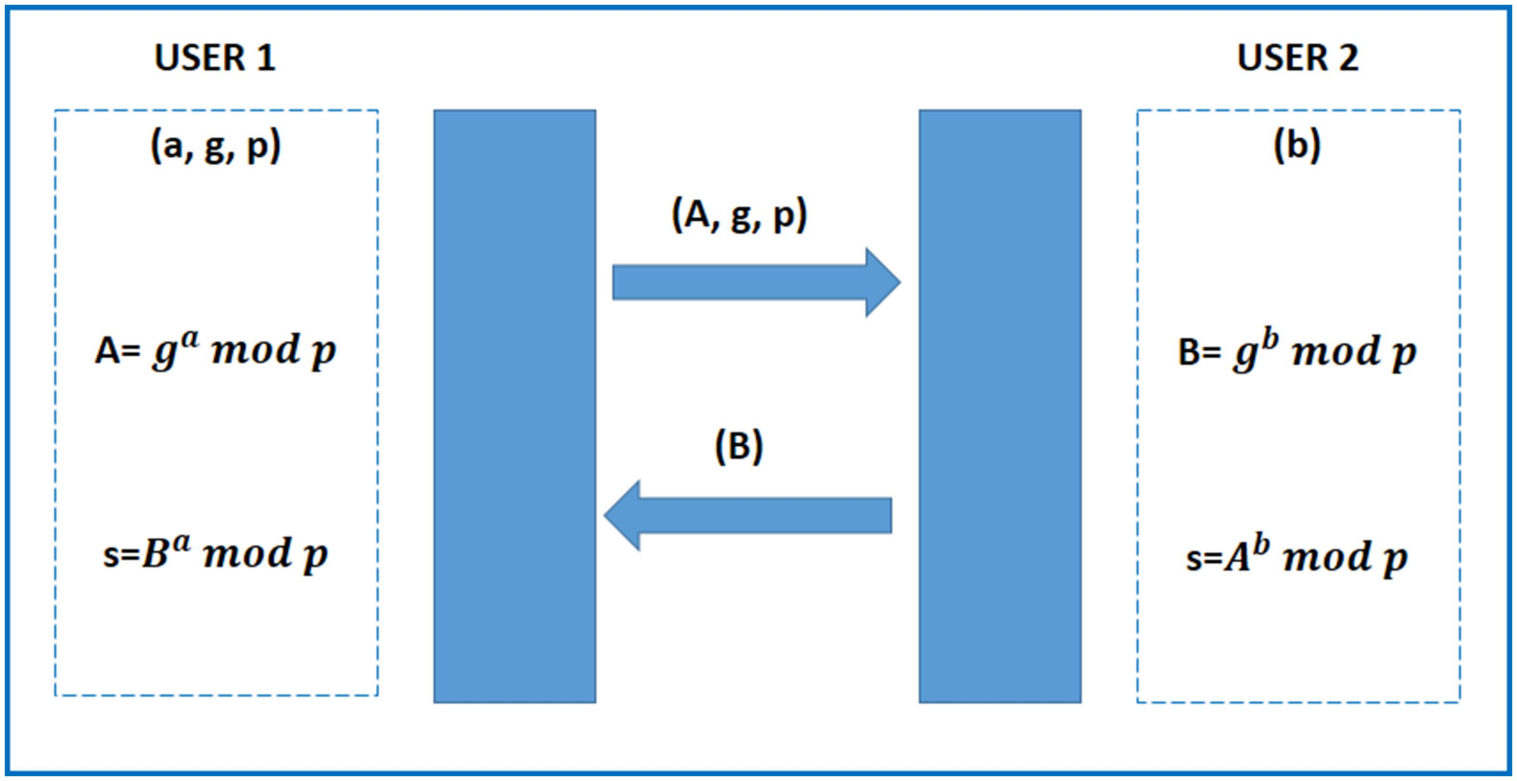
Diffie–Hellman key exchange protocol.

Now, both devices have arrived at the same shared secret keys without directly transmitting them over the insecure channel. This shared secret key can be used for subsequent symmetric encryption, ensuring the confidentiality and integrity of the communication between the IoT devices (Quist-Aphetsi and Xenya, 2019). The key exchange protocol of the DHE algorithm has been illustrated in Figure 4.

It’s important to note that while Diffie-Hellman provides a secure key exchange mechanism, it does not address other aspects of IoT security, such as authentication, authorization, and protection against various types of attacks. Therefore, a comprehensive IoT security strategy would likely involve a combination of cryptographic techniques, secure protocols, and best practices to address the specific requirements and challenges of IoT environments.

### 4.4 Twofish technology: enhancing data communication security

Twofish is a cryptographic algorithm that can handle plaintext of any size up to 128 bits. It is considered a candidate for the Advanced Encryption Standard (AES) as it functions as a symmetric key block cipher and takes inspiration from the Blowfish algorithm. The algorithm works on a Feistel network that divides the input into four subblocks (P0, P1, P2, P3) of 32 bits each. Additionally, four whitening keys (K0, K1, K2, K3) are used to increase the security of each block. The Feistel network structure includes a bijection process that ensures the safe transformation of the input. Each 32-bit block comes with a whitening key, which provides additional security for subentries. Whitening keys play an important role in improving the security of iterative block ciphers. The most common form of key whitening is the XOREncrypt XOR method. It uses a simple XOR operation before the first round of encryption and after the last round of encryption (Awan et al., 2022).

#### 4.4.1 Twofish algorithm

**Figure 5:**
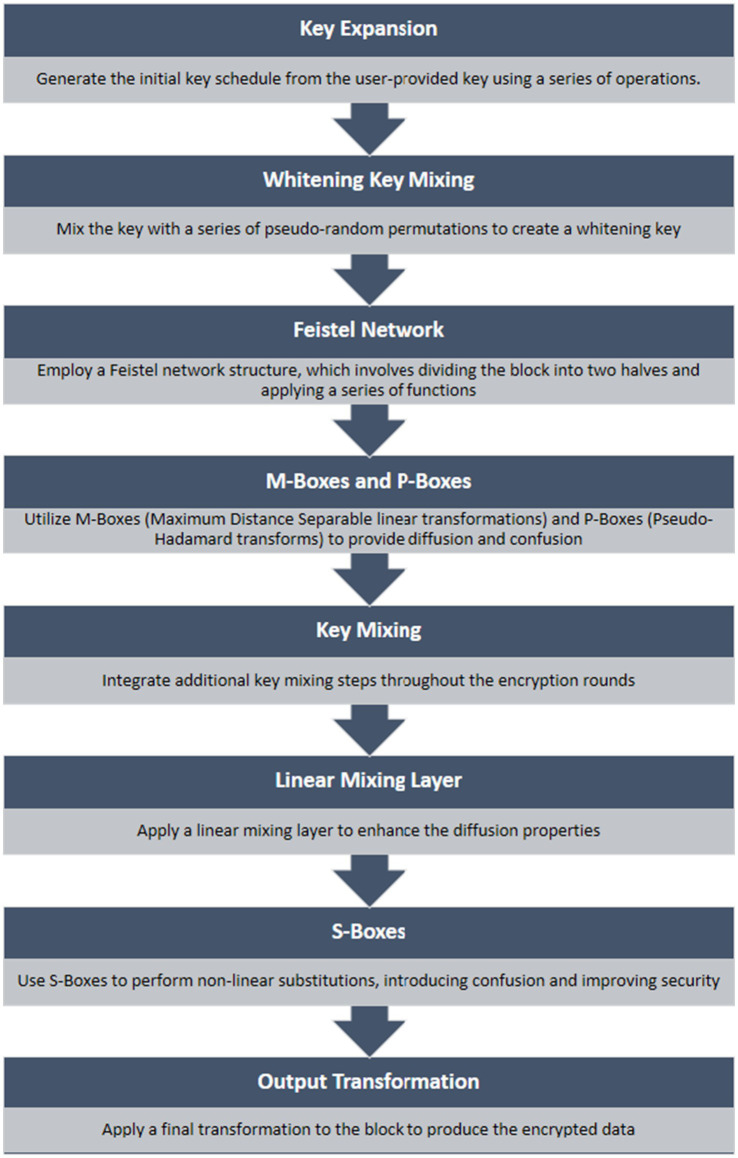
Flowchart of two-fish algorithm.

Twofish is a symmetric key block cipher algorithm designed for encryption (Haq et al., 2021). It was one of the five finalists of the Advanced Encryption Standard (AES) competition, which sought to establish a new encryption standard to replace the aging Data Encryption Standard (Smid, 2021). Though Twofish was not ultimately selected as the AES, it is still considered a highly secure and efficient encryption algorithm. Figure 5 illustrates the flowchart of the Twofish algorithm.

Here are the key features and aspects of Twofish technology for enhancing data communication security:

- *Symmetric key algorithm*: Twofish is a symmetric key algorithm, meaning the same key is used for both encryption and decryption. This requires secure key management to ensure the confidentiality of the communication.
- *Block cipher*: Twofish operates on fixed-size blocks of data (128 bits) and encrypts data in blocks. This is typical of block ciphers, and it means that data is processed in fixed-size chunks.
- *Key size*: Twofish supports key sizes of 128, 192, or 256 bits. The larger key sizes generally provide stronger security, but they may also require more computational resources.
- *Feistel network structure*: Twofish employs a Feistel network structure, a common design for block ciphers. The Feistel network alternates between dividing the data into two halves and applying a function that depends on the key.
- *Substitution-permutation network (SPN)*: Twofish uses a substitution-permutation network, combining substitution and permutation operations to achieve confusion and diffusion, important aspects of secure encryption.
- *Security and cryptanalysis*: Twofish has undergone extensive cryptanalysis and is considered secure. Its resistance to various types of attacks makes it suitable for use in applications requiring high levels of security.
- *Flexibility*: Twofish is designed to be flexible and can be implemented efficiently in both hardware and software. This makes it suitable for a variety of applications, including embedded systems and resource-constrained devices.
- *Open design*: Twofish’s design is open and has been subject to public scrutiny. Open designs allow for transparency, enabling security experts to review and analyze the algorithm for potential vulnerabilities.

When it comes to data communication security, Twofish can be used to encrypt sensitive information, providing confidentiality and integrity during transmission (Makarenko et al., 2020). It’s important to note that while encryption is a crucial aspect of secure communication, a comprehensive security strategy should also address other aspects, such as authentication, authorization, and secure key management. Additionally, the choice of encryption algorithm should consider the specific requirements and constraints of the communication environment.

### 4.5 Machine learning for IoT security

**Figure 6:**
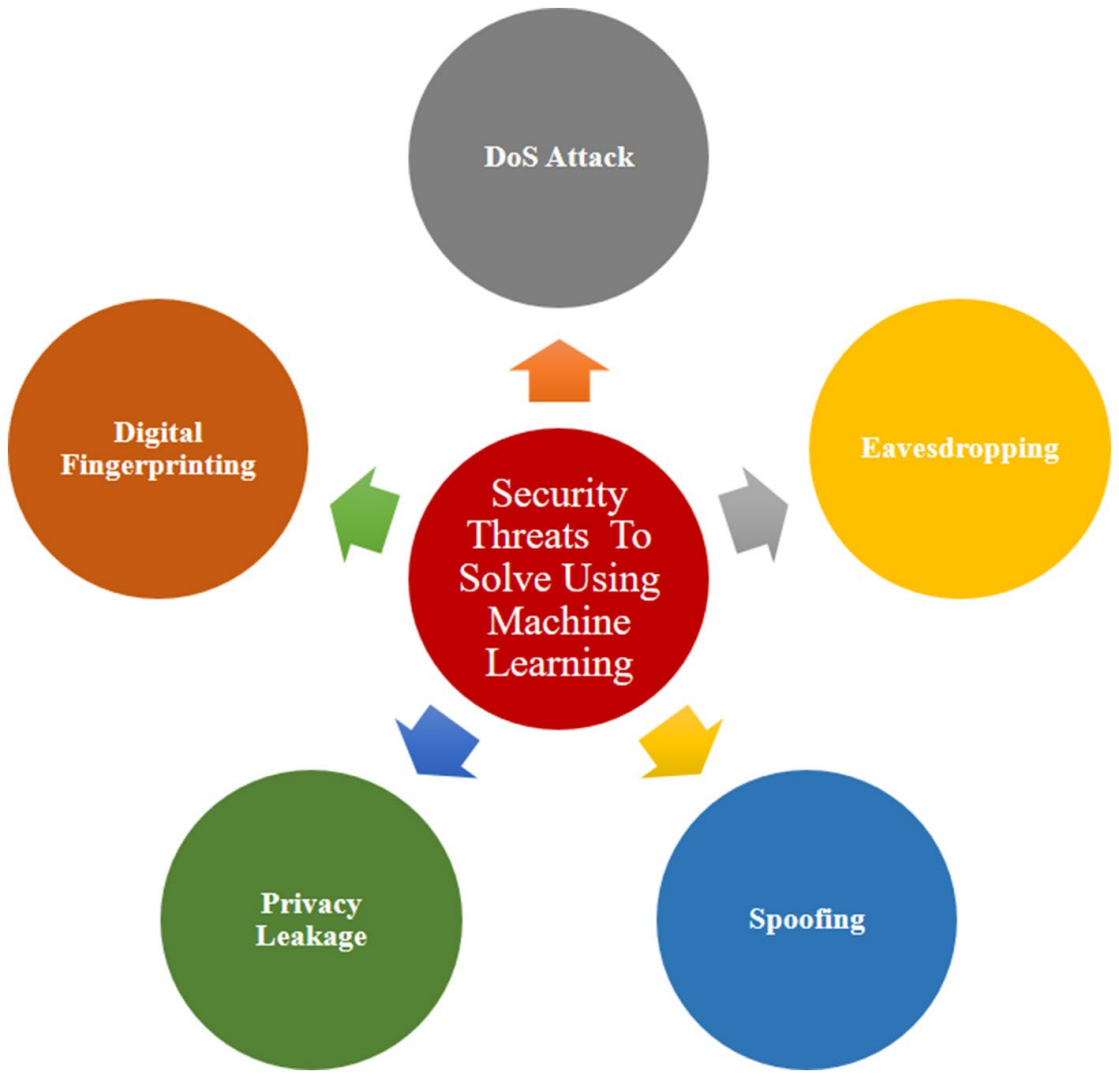
Various security threats which can be solved using ML.

The domain of machine learning (ML) has garnered substantial attention in recent years, with numerous domains incorporating ML into their development practices. Notably, ML is being actively employed in the realm of Internet of Things (IoT) security. It represents a great solution for safeguarding IoT devices against possible cyber-attacks, offering a distinct way to defend when compared to conventional methods. ML’s application in IoT security introduces a dynamic and adaptive layer that can enhance the resilience of devices, showcasing its potential to revolutionize the landscape of cybersecurity (Ahmad and Alsmadi, 2021). Figure 6 represents various security threats that can be solved using machine learning.

The options offered by ML to combat these security concerns are addressed further below:-.

a. DoS attack:

DoS attacks on IoT devices or emerging from IoT devices are a major issue. Implementing a Multi-Layer Perceptron (MLP)-based protocol developed to fortify networks against DoS attacks is one viable technique for thwarting attacks of this kind (Riahi et al., 2013). Using ML algorithms helps to improve deduction accuracy, which strengthens the security of IoT devices vulnerable to DoS attacks.

b. Eavesdropping:

Eavesdropping, in which adversaries intercept messages as they are being transmitted, is a significant risk. To counter this hazard, machine learning methods or non-parametric Bayesian methods might be used. Q-learning and Dyna-Q are two machine-learning approaches that can protect devices from eavesdropping. The evaluation of these schemes, which was carried out through various experiments and reinforcement learning (Khan and Salah, 2018).

c. Spoofing:

To counter spoofing attacks, a wide range of approaches, including Q-learning, Dyna-Q, SVM, Deep Neural Network models, incremental aggregated gradient, and distributed Frank Wolfe can be used. These techniques not only improve identification as well as classification precision but also contribute to lower mean error rates and instances of false alarms, protecting systems from spoofing attempts (Aldabbas and Amin, 2021).

d. Privacy leakage:

The collection of private data, encompassing health details, location data, or images, poses a threat to client privacy. To mitigate privacy leakage, the adoption of Privacy-preserving Scientific Computations (PPSC) becomes imperative. Additionally, a method known as Commodity Integrity Detection Algorithm (CIDA), rooted in the Chinese Remainder Theorem (CRT), has been suggested to install trust in IoT implementations and safeguard against privacy breaches.

e. Digital fingerprinting:

Digital fingerprinting stands out as a great solution for enhancing the security of IoT systems and fostering trust among end-users in various applications (Chowdhury and Abas, 2022). The widespread use of fingerprints for tasks such as unlocking smartphones, authorizing payments, and accessing vehicle and house doors attests to its popularity. With its attributes of less price and better reliability digital fingerprinting has emerged as a major biometric identification procedure. However, despite its advantages, the efficient implementation of digital fingerprinting in IoT faces several challenges. These include issues related to fingerprint classification, image enhancement, and feature matching. To address these challenges, several ML algorithms have been developed. Some notable algorithms include:

- Support Vector Machine (SVM): SVM is a flexible training approach that may be used for both linear and nonlinear classifications, such as principal component analysis, text categorization, speaker identification, and regression (Pisner and Schnyer, 2020). SVM optimizes the distance between the decision border and training patterns by creating a feature vector. This approach analyzes the fingerprint’s distinct patterns and permits matching based on the identified patterns. This technology examines the fingerprint’s distinct patterns and permits matching depending on the patterns detected.
- Artificial Neural Networks (ANN): ANN, is a widely used algorithm in machine learning that offers many advantages. ANN utilizes the digital values of various features as input for training. The backpropagation algorithm is employed to train the neural network, and fingerprint verification is carried out based on experiential values saved in the database. The role of machine learning is pivotal in the IoT landscape, aiming to protect all interconnected systems and devices. Machine learning algorithms are trained to detect anomalies or unwanted activities within IoT systems, thereby preventing information loss and mitigating potential issues. As the IoT ecosystem continues to grow, ongoing contributions and advancements in machine learning are essential to sustaining its security and development (Pacheco et al., 2020).

### 4.6 Edge computing for IoT security

Edge computing represents extensions of cloud computing, a technology widely embraced by diverse organizations. While these concepts—cloud, fog, and edge—may seem similar, they delineate distinct layers within the realm of IoT applications. The primary disparity among them lies in the location of intelligence and computational power. Cloud computing operates on an extensive scale, tasked with processing vast amounts of information. It typically resides at a considerable distance from end-users. To address the challenges inherent in cloud computing, edge computing emerges as a potential solution (Sha et al., 2020). Here, a compact edge server is strategically positioned in between the client and the cloud or fog. Unlike cloud computing, some processing activities occur at the edge server rather than solely within the cloud. The architecture of edge computing comprises edge devices, cloud servers, and fog nodes. In this framework, computation and analytical capabilities are decentralized, empowering the edge devices themselves. Devices within an implementation can establish a network, collaborating to compute information locally. This approach minimizes the need to transmit substantial amounts of data externally. Consequently, this enhances the security of IoT applications by reducing data exposure. Moreover, edge computing contributes to cost efficiency by curbing communication expenses. It achieves this by obviating the necessity to shuttle all information to the distant cloud. In summary, edge computing not only optimizes computational efficiency but also fortifies the security of IoT applications while promoting economic benefits through reduced communication overhead (Alwarafy et al., 2020).

#### 4.6.1 Edge computing for the improvement of security

Edge computing offers several solutions to address and mitigate security threats in IoT applications:

i. *Information breaches:*

All information is saved and processed locally within the device or local network in edge computing, eliminating the need for information to traverse between the originator and the processor. This approach minimizes the danger of information thefts and breaches, as the data is not in transit. In contrast, fog computing involves some shifting of information from devices to the fog layer, creating potential vulnerabilities that adversaries could exploit.

ii. *Information compliance issues:*

Some countries enforce very strict regulatory acts, like the European Union’s GDPR (General Data Protection Regulation), to control the movement of information across borders. Edge computing enables organizations to retain information within their geographical boundaries, ensuring compliance with information sovereignty laws and regulations. This localized approach helps address concerns related to information compliance.

iii. *Safety issues:*

Swift response times are essential to prevent safety issues, such as deploying airbags in a car in the event of an imminent crash. Edge computing allows sensors to analyze data locally, reducing reliance on sending all information to the cloud for decision-making. This ensures faster response times, mitigating the risk of injuries or death. Surveillance cameras, which are empowered by edge computing, may analyze anomalies locally and transmit concise information to information centers for quicker responses.

iv. *Bandwidth issues:*

IoT applications produce large volumes of data at very high rates, much of which is of quite low value. Transmitting all this information to the cloud incurs significant bandwidth costs and poses security challenges. Edge computing addresses bandwidth issues by performing information cleaning and aggregation at the edge nodes. Only the essential, concise information, if needed, is then transmitted to the cloud. This not only reduces bandwidth costs but also enhances the overall efficiency and security of information transmission.

#### 4.6.2 Challenge in edge layer

While edge computing offers a range of facilities to enhance the safety and performance of IoT applications, there exist numerous challenges. Edge devices encompass a variety of components such as sensors, RFID devices, actuators, tags, and embedded devices. The susceptibility of the edge layer to assaults in an IoT system poses a significant concern, as compromising the edge layer could jeopardize the entire system. The primary protocols for the edge layer, MQTT and COAP, lack a default security layer. While the option to include optional security layers exists, it creates extra processing and bandwidth overhead. Particular issues related to edge devices involve vulnerabilities to sleep deprivation attacks, outage attacks, and battery-draining attacks. Given that edge devices are usually resource-constrained, with their primary reliance on battery backup, a prominent and straightforward method of attacking them is to deplete their battery. For instance, an adversary might compel an edge device to engage in power-intensive computations. Finding a delicate equilibrium between keeping and processing information on the edge or in the cloud is crucial. Excessive information storage on the edge may overwhelm these devices, potentially impacting the entire application (Singh et al., 2020).

#### 4.6.3 Privacy protection

The decentralized nature of Edge computing introduces complexities in ensuring privacy, as data processing occurs closer to the data source, often at the edge of the network. This necessitates robust privacy protection measures to safeguard sensitive information, including data and location privacy. The key challenges and strategies associated with privacy protection in the Edge Layer of IoT deployments have been discussed below.

##### 4.6.3.1 Data privacy in the edge layer of IoT

Data privacy is paramount in IoT deployments, given the vast amounts of sensitive information generated and processed by Edge devices. In the Edge Layer, ensuring data privacy involves several key considerations:

- Data Encryption: Implementing strong encryption protocols to secure data transmission and storage is essential for protecting sensitive information from unauthorized access or interception. Encryption algorithms, such as Advanced Encryption Standard (AES) and Transport Layer Security (TLS), play a crucial role in safeguarding data confidentiality.
- Access Control: Enforcing strict access controls helps regulate data access within the Edge Layer, ensuring that only authorized personnel or systems can access sensitive information. Role-based access control mechanisms and multi-factor authentication protocols can be employed to restrict access based on user roles and permissions.
- Data Minimization: Collecting only the minimum amount of data necessary to fulfill specific purposes helps mitigate privacy risks associated with data storage and processing. By minimizing data collection, organizations can reduce the likelihood of privacy breaches and limit exposure to potential threats.
- Anonymization and Pseudonymization: Anonymizing or pseudonymizing personally identifiable information helps protect individual privacy while still enabling data analysis and utilization. Techniques such as data masking, tokenization, and hashing can be used to anonymize sensitive data, preventing the identification of individuals.
- User Consent and Transparency: Obtaining explicit consent from users before collecting their data and providing transparency regarding data collection practices are essential for ensuring compliance with privacy regulations and fostering trust. Organizations should communicate how data will be used, stored, and shared, allowing users to make informed decisions about their privacy.

##### 4.6.3.2 Location privacy in the edge layer of IoT

Location privacy is another critical aspect of privacy protection in IoT deployments, particularly concerning the collection and use of geolocation data by Edge devices. Protecting location privacy involves addressing the following challenges:

i. Location Masking: Minimizing the collection of precise location data and utilizing techniques such as location aggregation or masking help preserve individual anonymity and prevent the unauthorized disclosure of sensitive location information.
ii. Geofencing: Implementing geofencing mechanisms enables organizations to define virtual boundaries around sensitive locations, restricting data collection and transmission within designated areas. Geofencing helps mitigate the risk of exposing sensitive location information and ensures compliance with privacy regulations.
iii. Anonymization of Location Data: Anonymizing location data by aggregating it at a higher level of granularity or removing identifying information helps prevent the identification of individuals or devices. Anonymization techniques, such as spatial cloaking and k-anonymity, can be employed to protect location privacy while still enabling meaningful data analysis.
iv. Secure Transmission: Ensuring the secure transmission of location data using encryption and robust security protocols is crucial for protecting against interception or unauthorized access. Secure transmission mechanisms, such as secure sockets layer (SSL) and virtual private networks (VPNs), help maintain the confidentiality and integrity of location information during transit.
v. Granular User Control: Providing users with granular control over their location data, including the ability to specify access preferences and usage permissions, empowers individuals to manage their privacy preferences effectively. Granular user control enhances transparency and accountability in location data handling practices, fostering trust and compliance with privacy regulations.

##### 4.6.3.3 Challenges and future directions

While significant progress has been made in addressing privacy concerns in the Edge Layer of IoT, several challenges and future research directions remain:

i. Standardization: The lack of standardized privacy frameworks and protocols for Edge computing poses challenges in ensuring interoperability and consistency across diverse IoT ecosystems. Future research efforts should focus on developing standardized privacy frameworks tailored to the unique requirements of the Edge Layer.
ii. Scalability: The scalability of privacy-preserving techniques, particularly concerning resource-constrained Edge devices, presents challenges in deploying robust privacy protection mechanisms at scale. Future research may explore lightweight encryption algorithms and optimization techniques to enhance the scalability of privacy solutions in the Edge Layer.
iii. Emerging Technologies: The emergence of new technologies, such as edge artificial intelligence (AI) and blockchain, introduces new opportunities and challenges for privacy protection in the Edge Layer. Future research directions may involve exploring the integration of AI-based privacy-enhancing techniques and decentralized privacy-preserving mechanisms using blockchain technology.
iv. Ethical Considerations: Addressing the ethical implications of data collection and processing at the Edge is essential for ensuring responsible and ethical use of IoT technologies. Future research efforts should prioritize ethical considerations, including fairness, transparency, and accountability, in the design and deployment of privacy protection mechanisms in the Edge Layer.

## 5 Risk methodologies and standards for IoT

Several risk methodologies and standards can be useful for managing risks associated with IoT (Internet of Things) deployments. Some of these include:

i. *ISO/IEC 27005:* This standard provides guidelines for information security risk management. It can be applied to assess and manage risks associated with IoT deployments, helping organizations identify and mitigate potential threats (Danielis et al., 2020).
ii. *NIST Cybersecurity Framework:* Developed by the National Institute of Standards and Technology (NIST) in the United States, this framework provides guidance on managing and reducing cybersecurity risks across various sectors, including IoT. It offers a structured approach to identifying, protecting, detecting, responding to, and recovering from cyber threats (Webb and Hume, 2018).
iii. *OWASP IoT Top 10:* The Open Web Application Security Project (OWASP) publishes a list of top security concerns specific to IoT devices and applications. It includes vulnerabilities and risks commonly found in IoT deployments, helping organizations prioritize their security efforts (Ferrara et al., 2021).
iv. *ENISA IoT Security Baseline:* The European Union Agency for Cybersecurity (ENISA) has developed guidelines for IoT security, including risk assessment and management practices. These guidelines aim to help organizations enhance the security of their IoT deployments (Khurshid et al., 2022).
v. *IEC 62443:* This series of standards, developed by the International Electrotechnical Commission (IEC), addresses the security of industrial automation and control systems, including those used in IoT deployments. It provides guidelines for assessing and managing cybersecurity risks in industrial environments (Hassani et al., 2021).
vi. *FAIR (Factor Analysis of Information Risk):* FAIR provides a quantitative framework for analyzing and assessing information security risks. While not specific to IoT, it can be adapted to evaluate the financial impact of risks associated with IoT deployments (Rana et al., 2024).

## 6 Future scope

The rapid evolution of the Internet of Things (IoT) landscape necessitates ongoing research and development efforts to ensure its secure and efficient integration across various sectors. Building upon the foundation laid by existing studies, future research in IoT security can explore several promising avenues to address emerging challenges and enhance the resilience of IoT ecosystems.

i. Advanced Threat Detection and Mitigation Techniques: Continued advancements in machine learning and artificial intelligence can be leveraged to develop more sophisticated threat detection and mitigation techniques tailored specifically for IoT environments. Research in this area can focus on refining anomaly detection algorithms, enhancing predictive maintenance models, and developing dynamic response mechanisms to counter evolving cyber threats effectively.
ii. Integration of Emerging Technologies: With the emergence of new technologies such as quantum computing and homomorphic encryption, future research can explore their applicability in fortifying IoT security. Investigating how quantum-resistant cryptographic algorithms can safeguard IoT communications and data integrity, and exploring the potential of homomorphic encryption for secure and privacy-preserving data processing within IoT networks are areas ripe for exploration.
iii. Privacy-Preserving Solutions: As the collection and processing of sensitive data become pervasive in IoT applications, there is a growing need for privacy-preserving solutions. Future research can delve into techniques such as differential privacy, secure multiparty computation, and federated learning to enable secure data sharing and collaborative analysis while preserving individual privacy rights.
iv. Standardization and Interoperability: Establishing robust standards and protocols for IoT security is crucial to ensuring interoperability and compatibility across diverse IoT ecosystems. Future research efforts can focus on developing standardized security frameworks, protocols, and certification mechanisms to promote uniform security practices and facilitate seamless integration of IoT devices and platforms.
v. Resilience Against Physical Attacks: In addition to cybersecurity threats, IoT systems are vulnerable to physical attacks such as tampering, tamper-resistant mechanisms, and secure hardware implementations. Future research can explore innovative approaches to enhance the physical security of IoT devices, including the integration of hardware-based security features, secure bootstrapping procedures, and tamper-evident packaging solutions.
vi. User-Centric Security Solutions: Empowering end-users with tools and resources to actively participate in securing IoT devices and networks is essential. Future research can focus on developing user-friendly security interfaces, educational resources, and incentivization mechanisms to promote security awareness and encourage proactive risk mitigation practices among IoT stakeholders.
vii. Regulatory and Policy Considerations: As IoT adoption continues to accelerate, there is a pressing need for comprehensive regulatory frameworks and policies to govern the responsible deployment and operation of IoT systems. Future research can explore the socio-economic implications of IoT security regulations, assess regulatory compliance challenges, and propose strategies for harmonizing global standards to ensure a cohesive and effective regulatory landscape.

## 7 Conclusion

In conclusion, this survey paper has provided a comprehensive overview of the security risks and challenges inherent in Internet of Things (IoT) ecosystems, offering insights into the diverse array of threats that can compromise the integrity and confidentiality of data transmitted and processed by IoT devices. By examining the vulnerabilities at various layers of the IoT architecture, including the sensing layer, network layer, middleware layer, gateways, and application layer, we have highlighted the critical importance of implementing robust security measures to mitigate potential risks and enhance the overall safety posture of IoT deployments. Through a detailed analysis of existing security issues and potential research directions, this paper has underscored the need for continuous innovation and collaboration in the field of IoT security to address emerging threats and vulnerabilities effectively. By exploring cutting-edge technologies such as machine learning, blockchain, and edge computing as potential solutions to bolster IoT security, we have laid the groundwork for future research aimed at fortifying the resilience of IoT ecosystems against evolving cyber threats. As IoT continues to evolve and expand its reach across diverse sectors, stakeholders must prioritize security considerations and adopt proactive measures to safeguard sensitive information and ensure the reliable operation of connected devices. By fostering a culture of security awareness and knowledge sharing, we can collectively work toward creating a safer and more secure digital landscape for IoT applications to thrive and deliver on their transformative potential.

## 8 Summary

The Internet of Things (IoT) is poised to revolutionize various sectors, promising unprecedented connectivity and efficiency. Defined as a network of physical objects or “things” embedded with sensors, actuators, RFID tags, and other technologies for communication over the internet, IoT is expected to bring about significant advancements in healthcare, agriculture, transportation, industrial automation, smart cities, and smart governance. This transformative concept holds the potential to enhance societal services with minimal effort. However, the connectivity inherent in IoT introduces a spectrum of security threats, encompassing active, passive, and physical risks to the system. The challenges are further compounded by the resource constraints of IoT devices, rendering traditional cryptosystems impractical. Additionally, these systems are vulnerable to physical attacks. The balance between reaping the benefits of IoT and implementing robust security measures becomes imperative to harness its potential while mitigating associated risks. The architecture of IoT comprises five layers: Sensing Layer, Network Layer, Middleware Layer, Gateway Layer, and Application Layer. Each layer leverages diverse technologies, contributing to the overall functionality and effectiveness of an IoT system. The Sensing Layer, linked with physical sensors and actuators, faces security issues such as sensor tampering, false code injection, side-channel attacks, eavesdropping, and increased power consumption. The Network Layer, responsible for transmitting sensor data, is susceptible to phishing attacks, DDoS attacks, and routing attacks. The Middleware Layer, a link between the Network and Application Layers, encounters challenges like man-in-the-middle attacks, SQL injection, signature wrapping, and cloud malware injection. The Gateway Layer, connecting users and cloud services, must address secure onboarding, end-to-end encryption, and firmware update security. The Application Layer, serving end-users, faces threats like information theft, access control attacks, service interruption attacks, false code-sending attacks, sniffing attacks, and reprogramming attacks. To address these security challenges, various solutions are proposed. Blockchain technology enhances transparency, security, and trust in IoT systems by using a distributed, decentralized, and shared ledger. Fog computing addresses real-time services, transient storage, information dissemination, and decentralized computation, bringing computational resources closer to the edge of the network. Machine learning offers proactive security measures such as anomaly detection, intrusion detection, predictive maintenance, behavioral analysis, and security threat intelligence. Twofish technology, a symmetric key block cipher, and the Diffie-Hellman encryption technique contribute to securing communication channels and data storage in IoT devices.

In conclusion, a holistic approach that combines these diverse security solutions is necessary to effectively address the intricate and evolving security landscape of the Internet of Things. Balancing the benefits of IoT with robust security measures is imperative to unlock its potential while mitigating associated risks. As IoT continues to proliferate and shape the future, the collaborative efforts of industry stakeholders, researchers, and policymakers are crucial to ensuring a secure and resilient IoT ecosystem.

## Author contributions

SS: Conceptualization, Methodology, Writing – original draft, Writing – review & editing. KM: Conceptualization, Methodology, Writing – review & editing, Writing – original draft.
